# Predictive models for intestinal obstruction: from clinical scores to artificial intelligence

**DOI:** 10.3389/fsurg.2026.1853765

**Published:** 2026-07-15

**Authors:** Zehao Liu, Li He, Qiangqiang Zhang, Runyu Chen, Zhonghu Li

**Affiliations:** 1 School of Medicine, Wuhan University of Science and Technology, Wuhan, China; 2 General Surgery Department, Central Theater Command General Hospital of the Chinese People’s Liberation Army, Wuhan, China

**Keywords:** artificial intelligence, computed tomography, intestinal obstruction, machine-learning, nomogram, prediction model, small bowel obstruction, surgical decision-making

## Abstract

**Background and objective:**

Intestinal obstruction is a common surgical emergency in which delayed recognition of strangulation, ischemia, or failure of non-operative management can lead to bowel necrosis, sepsis, and death. Prediction models have been developed for several related but distinct tasks, including diagnosis of obstruction, prediction of urgent surgery, prediction of strangulation or irreversible ischemia, and prediction of failure of conservative treatment. This review critically summarizes these models and clarifies their clinical scope, validation status, and translational limitations.

**Methods:**

PubMed was searched for studies published from database inception to October 31, 2025, with the initial search performed during manuscript preparation on December 1, 2025 and the final update search conducted on June 18, 2026. Search terms were related to intestinal obstruction, small bowel obstruction, prediction model, nomogram, scoring system, artificial intelligence, machine learning, deep learning, and computed tomography. Eligible articles reported or discussed predictive, diagnostic, or decision-support models for intestinal obstruction. We excluded papers without model-related content, non-clinical mechanistic studies unless used to explain modeling rationale, case reports, and articles not providing sufficient methodological or performance information. Because the evidence was heterogeneous in population, endpoint, modality, and design, the review was synthesized narratively rather than meta-analyzed.

**Key findings:**

Conventional clinical scores remain attractive because they are transparent, inexpensive, and quickly calculable, but their performance varies across endpoints and settings. CT-integrated models improve anatomical and ischemic risk assessment but depend on imaging availability and reader expertise. Machine-learning and deep-learning models, including multimodal systems combining electronic health records and imaging, have reported high discrimination in selected datasets; however, many studies remain retrospective, single-center, and incompletely externally validated. Therefore, AUC values across studies should not be interpreted as directly comparable evidence of superiority.

**Conclusions:**

The field is moving from static, single-modality scores toward dynamic, multimodal decision-support systems. The most immediate research priorities are prospective multicenter validation, standardized endpoint definitions, calibration and decision-curve reporting, explainability, fairness assessment, privacy-preserving data sharing, and workflow integration in emergency surgical care.

## 1 Introduction

Intestinal obstruction is a frequent cause of acute abdomen and emergency surgical consultation. The clinical challenge is not only to diagnose obstruction, but also to identify patients who require urgent intervention because of strangulation, irreversible ischemia, bowel necrosis, or likely failure of conservative treatment (1–6). Delayed surgery may increase morbidity and mortality, whereas unnecessary surgery exposes patients to avoidable complications. Risk stratification tools are therefore clinically important.

Prediction models for intestinal obstruction have expanded rapidly. Early models used readily available bedside variables, such as age, abdominal signs, ascites, and gastrointestinal decompression volume (7). Later models incorporated CT features and laboratory variables to improve the recognition of strangulation and ischemia (8–14). More recently, machine-learning and deep-learning approaches have been applied to abdominal radiographs, CT images, and electronic health-record data (15–21).

The novelty of this review is threefold. First, it separates prediction models according to clinical task, because models predicting surgery, strangulation, ischemia, non-operative treatment failure, or radiographic obstruction answer different clinical questions. Second, it proposes a three-generation conceptual framework that distinguishes bedside clinical scores, CT-integrated multidimensional models, and AI-enabled multimodal systems. Third, it evaluates reported performance in light of methodological heterogeneity, external validation, clinical workflow feasibility, explainability, and generalizability rather than ranking models by AUC alone.

This review is intended for emergency surgeons, gastrointestinal surgeons, radiologists, emergency physicians, and clinical AI researchers who need a practical and critical overview of prediction tools for intestinal obstruction.

### 1.1 Focused review questions

1. Which predictive models have been developed for intestinal obstruction and what clinical endpoints do they target?
2. How have model inputs evolved from clinical variables to CT imaging and multimodal AI?
3. How should performance be interpreted given heterogeneity in population, endpoint, validation, and study design?
4. What methodological and translational steps are required before AI-based models can be used safely in routine emergency surgical workflows?

## 2 Review methodology

### 2.1 Search strategy and reporting framework

This review was designed as a narrative review with a semi-systematic PubMed-based search strategy. The report was revised with reference to the SANRA principles for narrative reviews and, where applicable, PRISMA concepts for transparent search reporting. PubMed was used as the primary bibliographic database. In addition, the reference lists of eligible articles and relevant reviews were manually screened to identify additional studies. The PRISMA-style flow diagram was used only to improve search transparency rather than to claim full compliance with PRISMA systematic-review methodology.

The initial literature search was performed during manuscript preparation on December 1, 2025, and an updated final PubMed search was conducted on June 18, 2026. To maintain a predefined and reproducible evidence window, eligible records were restricted to studies published from database inception to October 31, 2025. Reference lists of eligible articles and relevant reviews were also manually screened, and any additional studies were included only if they met the same eligibility criteria and publication cutoff. Recent methodological or background references outside this evidence window, if cited, were used only for contextual discussion and were not counted among the included prediction-model studies.

The following PubMed search string was used:

(“intestinal obstruction”[Title/Abstract] OR “bowel obstruction”[Title/Abstract] OR “small bowel obstruction”[Title/Abstract] OR “adhesive small bowel obstruction”[Title/Abstract]) AND (“prediction model”[Title/Abstract] OR “predictive model”[Title/Abstract] OR “risk score”[Title/Abstract] OR “scoring system”[Title/Abstract] OR nomogram[Title/Abstract] OR “decision support”[Title/Abstract] OR diagnosis[Title/Abstract] OR prediction[Title/Abstract]) AND (“computed tomography”[Title/Abstract] OR CT[Title/Abstract] OR radiomics[Title/Abstract] OR “artificial intelligence”[Title/Abstract] OR “machine learning”[Title/Abstract] OR “deep learning”[Title/Abstract] OR CNN[Title/Abstract] OR “electronic health record”[Title/Abstract] OR EHR[Title/Abstract]).

The PubMed search identified 2,537 records. No duplicate records were removed because only one bibliographic database was used. After title and abstract screening, 2,247 records were excluded because they were not relevant to intestinal obstruction, did not contain prediction-model, diagnostic-model, scoring-system, nomogram, CT-based, radiomics, machine-learning, or deep-learning content, or were case reports, technique-only papers, animal studies, or non-clinical studies. A total of 290 reports were sought for retrieval, of which 8 could not be retrieved. Therefore, 282 full-text reports were assessed for eligibility.

**Figure 1:**
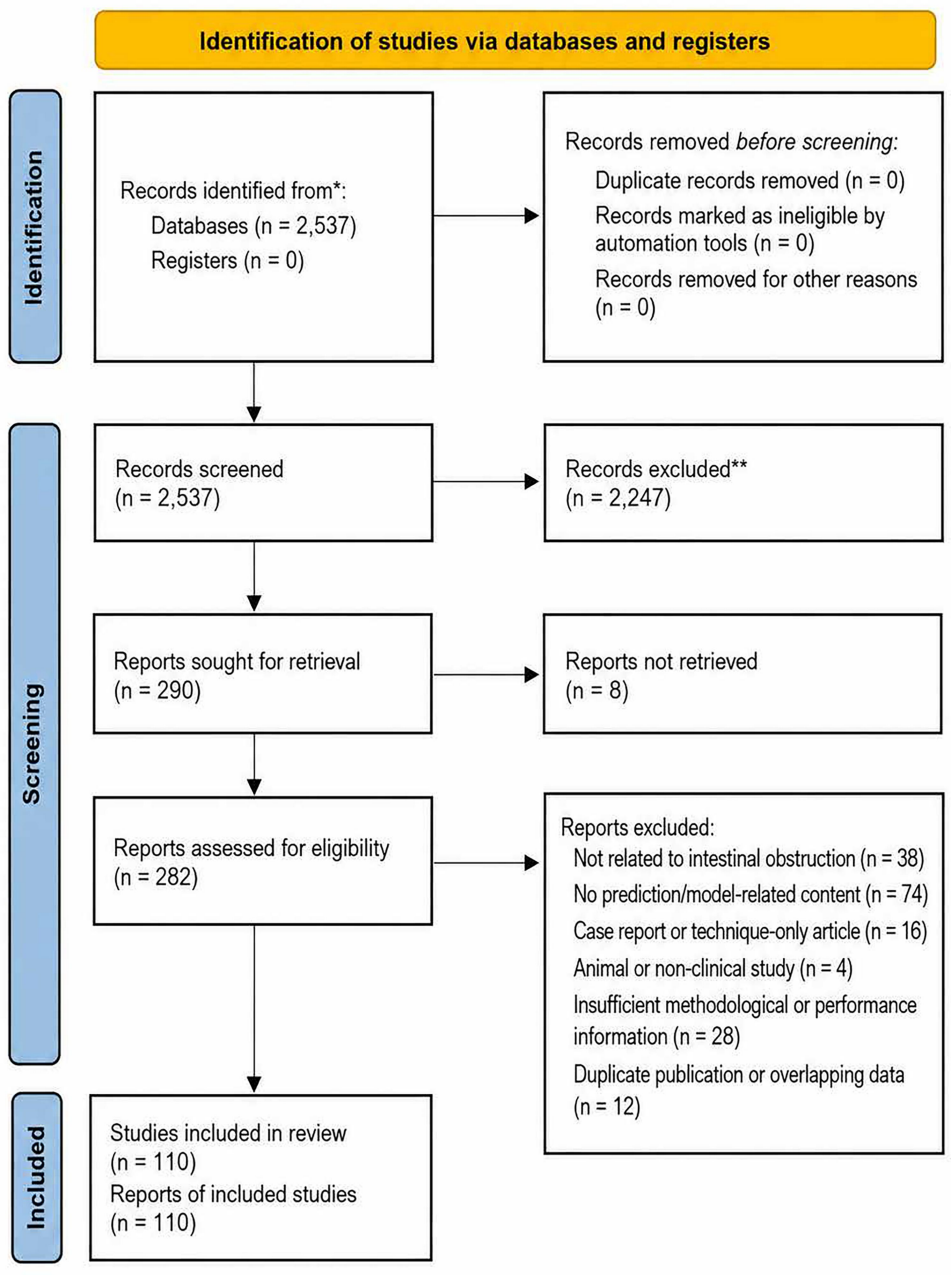
PRISMA-style search-transparency flow diagram for the PubMed-based semi-systematic literature search and study selection. ^*^PubMed was used as the primary bibliographic database. ^**^Records were excluded after title and abstract screening because they were not relevant to intestinal obstruction, did not contain prediction-model, diagnostic-model, scoring-system, nomogram, CT-based, radiomics, machine-learning, or deep-learning content, or were case reports, technique-only papers, animal studies, or non-clinical studies.

During full-text assessment, 172 reports were excluded for the following reasons: not related to intestinal obstruction, no prediction/model-related content, case report or technique-only article, animal or non-clinical study, insufficient methodological or performance information, or duplicate publication/overlapping data. Finally, 110 studies were included in the narrative synthesis. The study-selection process is shown in Figure 1.

### 2.2 Eligibility criteria

Studies were eligible if they involved adult or mixed clinical populations with intestinal obstruction, especially small bowel obstruction or adhesive small bowel obstruction, and reported or discussed clinical scores, nomograms, CT-based models, radiomics, machine-learning, deep-learning, or multimodal prediction systems. Eligible outcomes included diagnosis of obstruction, need for urgent surgery, strangulation, bowel ischemia or necrosis, bowel resection, and failure of conservative management. We excluded case reports, technique-only articles, purely animal or cellular studies unless used only to explain pathophysiological rationale, duplicate or overlapping publications, and studies without sufficient methodological or performance information. The detailed inclusion and exclusion criteria are summarized in Table 1.

**Table 1:** Eligibility criteria for study selection.

Domain	Inclusion criteria	Exclusion criteria
Population	Adult or mixed clinical populations with intestinal obstruction, especially small bowel or adhesive small bowel obstruction.	Purely experimental animal or cellular studies unless used only to explain pathophysiological rationale.
Model type	Clinical scores, nomograms, CT-based models, radiomics, machine-learning, deep-learning, or multimodal prediction systems.	Articles without model-related content or without a clear diagnostic/prognostic decision task.
Outcomes	Diagnosis of obstruction, urgent surgery, strangulation, bowel ischemia/necrosis, bowel resection, or failure of conservative management.	Case reports, technique-only papers, or studies lacking sufficient outcome or performance information.
Evidence type	Original studies, guidelines, consensus statements, and high-quality reviews relevant to model development, validation, or implementation.	Duplicated publications, abstracts without enough methodological detail, or articles not accessible for evaluation.

### 2.3 Study selection, data extraction, and synthesis

Two reviewers independently screened titles and abstracts. Potentially eligible full texts were then assessed independently by the same reviewers. Disagreements were resolved through discussion, and when consensus could not be reached, a senior author adjudicated. Data extraction was performed using a predefined form and checked by a second reviewer.

For predictive-model studies, extracted information included population, study design, sample size, target outcome, input modality, modeling method, validation strategy, discrimination, calibration, and clinical-utility assessment. Because studies differed substantially in endpoint definition, case mix, imaging availability, and validation design, quantitative pooling was not performed. Instead, evidence was grouped by clinical task and model generation, with emphasis on methodological comparability and clinical applicability.

For representative original prediction-model studies, a simplified PROBAST-based assessment was performed to evaluate risk of bias and applicability (22). The assessment focused on the four PROBAST domains: participants, predictors, outcome, and analysis. Each domain was judged as low, high, or unclear risk of bias according to the information reported in the original articles. Applicability concerns were considered in relation to the target population, predictors, and clinical outcomes addressed in this review. Because this article was designed as a narrative review with a semi-systematic PubMed-based search strategy rather than a full systematic review, PROBAST assessment was applied to representative original model-development or validation studies rather than to all background reviews, guidelines, mechanistic articles, and general artificial-intelligence methodology papers.

## 3 Clinical scope: distinct prediction tasks

A central problem in the literature is that “predictive model for intestinal obstruction” can refer to several different decision tasks. These endpoints should not be compared as if they were interchangeable (Table 2).

**Table 2:** Distinct clinical prediction tasks in intestinal obstruction.

Clinical task	Typical endpoint	Clinical decision supported	Why direct AUC comparison is problematic
Detection of obstruction	Radiographic or clinical diagnosis of bowel obstruction	Triage, radiology support, confirmation of suspected obstruction	Often evaluates image recognition rather than surgical urgency or ischemia.
Need for surgery	Operative intervention during admission or early hospitalization	Early operative versus conservative management	Surgery depends on local practice, surgeon preference, patient frailty, and resource availability.
Strangulation or ischemia	Compromised bowel perfusion, strangulated obstruction, or intestinal ischemia	Emergency surgery and intensified monitoring	Endpoint definitions vary; some include reversible ischemia, whereas others require necrosis.
Irreversible ischemia/bowel resection	Necrosis or resection confirmed intraoperatively	Predicting non-viable bowel and resection risk	Represents a more severe and less frequent endpoint; sensitivity is clinically critical.
Failure of conservative treatment	Need for surgery after initial non-operative management	Patient selection for water-soluble contrast protocols and observation	Depends on treatment protocol duration and institutional thresholds for surgery.

## 4 Pathophysiological rationale for prediction modeling

Intestinal obstruction is a dynamic process. Luminal blockage causes proximal accumulation of gas and fluid, bowel dilatation, increased intraluminal pressure, venous and lymphatic congestion, intestinal wall edema, impaired mucosal perfusion, and eventually ischemia (23). These changes explain why many models use variables such as abdominal pain duration, peritoneal irritation, fever, leukocytosis, CRP, lactate, bowel-wall thickening, reduced enhancement, ascites, mesenteric edema, closed-loop signs, and whirl signs (24–29).

**Figure 2:**
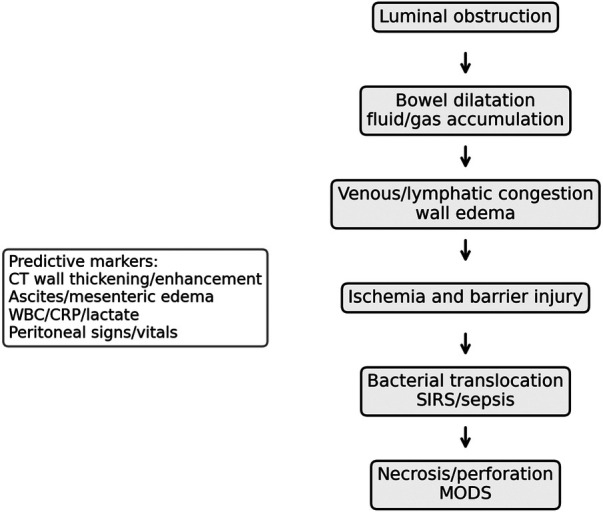
Pathophysiological cascade linking obstruction progression to candidate predictive markers.

The pathophysiological cascade also clarifies why single markers rarely provide reliable prediction. For example, vomiting and third-space fluid loss can produce electrolyte abnormalities and hypovolemia, whereas strangulation may produce systemic inflammation, bacterial translocation, metabolic acidosis, septic shock, and MODS (30–32). A clinically useful model should therefore integrate signals from symptoms, physical signs, laboratory tests, and imaging rather than rely on a single late manifestation (33). This pathophysiological cascade is summarized in Figure 2.

## 5 Conceptual framework: three generations of predictive models

**Figure 3:**
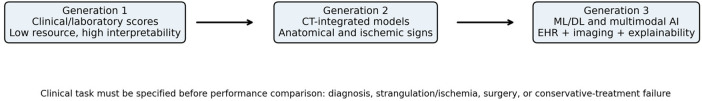
Conceptual evolution of predictive models for intestinal obstruction.

The three-generation framework is organizational rather than chronological. The conceptual evolution of predictive models for intestinal obstruction is shown in Figure 3. A model is classified by its dominant data source and intended mode of clinical use. Some papers published recently still belong to the first or second generation if they use transparent regression scores or CT-based variables rather than automated AI pipelines (Table 3).

**Table 3:** Three-generation conceptual framework for predictive models in intestinal obstruction.

Generation	Defining features	Typical strengths	Typical limitations	Representative examples
First generation: clinical/laboratory scores	Bedside symptoms, physical signs, vital signs, and routine laboratory tests; usually logistic regression or simple scoring.	Low cost, high interpretability, rapid bedside use, feasible in resource-limited settings.	Limited anatomical information; performance may fall when case mix changes; internal validation is often insufficient.	Komatsu (7), BAR-N (34).
Second generation: CT-integrated multidimensional models	Clinical and laboratory variables combined with CT signs such as ascites, closed loop, wall thickening, reduced enhancement, mesenteric edema, and whirl sign.	Better link to ischemic anatomy and surgical findings; interpretable to surgeons and radiologists.	Requires CT availability and standardized interpretation; contrast-enhanced CT may be limited by allergy or renal dysfunction.	Schwenter (11), Huang (9), Xu (13), STRISK/NOFA (14).
Third generation: ML/DL and multimodal AI	Algorithms such as LASSO, XGBoost, CNN, 3D CNN, transfer learning, radiomics, or EHR-image fusion; may include explainability layers.	Can model nonlinear interactions, image patterns, and multimodal data; potential for real-time decision support.	Risk of overfitting, bias, limited external validation, computational burden, weak interpretability, and workflow barriers.	Kim (15), Matsuda (18), Oh (19), Wang (16).

## 6 Traditional and CT-integrated scoring systems

Traditional models use variables that clinicians can obtain early during emergency evaluation. Their major advantage is transparency: the clinical meaning of each variable is usually intuitive. However, their apparent performance must be interpreted in the context of endpoint definition, sample size, and validation strategy.

The Komatsu model used age, ascites, and high decompression output to predict the need for surgery in patients initially managed conservatively. The Schwenter clinicoradiological score combined clinical, laboratory, and CT features and reported strong discrimination for strangulated small bowel obstruction. Huang et al. developed a model including fever, peritoneal irritation, leukocytosis, intestinal-wall thickening, and ascites, reporting an AUC of 0.935 for strangulated obstruction. More recent models such as BAR-N, Xu et al., STRISK, and NOFA attempted to improve usability, external validation, or endpoint specificity.

Calibration and clinical utility should be reported more consistently. Hosmer-Lemeshow testing alone is insufficient because it is sensitive to sample size and does not quantify absolute prediction error. Future studies should report calibration plots, calibration intercept and slope, Brier score, and decision-curve analysis. Decision-curve analysis is especially relevant because the net benefit of a model depends on the threshold at which a surgeon would operate or intensify monitoring (35–37) (Tables 4, 5).

**Table 4:** Summary of representative prediction-model studies and reported performance.

Model	Design/sample	Endpoint	Modality	Validation	Reported performance	Clinical interpretation
Komatsu (7)	Retrospective; adhesive SBO initially treated conservatively	Need for surgery	Clinical + ascites + decompression output	Model development; limited external evidence	Simple model; performance varies by setting	Useful when conservative management is being considered, but outcome reflects operative decision-making as well as biology.
Schwenter (11)	Clinicoradiological score	Strangulated SBO/necrosis risk	Clinical, CRP/WBC, abdominal fluid, CT enhancement	Limited external validation	AUC reported around 0.87; high risk at score >=4	Clinically intuitive but depends on CT interpretation and severe endpoint prevalence.
Huang (9)	Prediction model	Strangulated SBO	Temperature, peritoneal signs, WBC, CT wall thickening, ascites	Mainly development cohort	AUC 0.935	Strong reported discrimination, but comparability is limited by single-outcome design.
BAR-N (34)	Single-center retrospective; *n* = 453	Strangulated bowel obstruction	Rebound tenderness, BMI, neutrophil percentage, AST	Internal validation only	Training AUC 0.784; validation AUC 0.750; specificity high but sensitivity low	Practical early bedside tool; low sensitivity may limit rule-out use.
Xu (13)	Single-center retrospective; *n* = 281	Strangulated bowel obstruction/transmural necrosis	Symptoms, signs, electrolytes, BUN, CT score	External validation reported	Training AUC 0.857; validation AUC 0.910	Multidimensional and clinically rich, but calculation is relatively complex.
STRISK/NOFA (14)	Prospective multicenter; *n* = 481	STRISK: strangulation; NOFA: failure of non-operative treatment	Clinical history, signs, CT mesenteric changes, loop/stool signs	External validation cohort	STRISK AUC 0.860/0.907; NOFA AUC 0.751/0.751	Methodologically stronger because endpoints are separated and externally validated.

**Table 5:** Simplified PROBAST-based assessment of representative prediction-model studies.

Model	Clinical task	Participants	Predictors	Outcome	Analysis	Overall risk of bias
Komatsu et al. (7)	Need for surgery after initial conservative management	Unclear	Low	Unclear	High	High
Schwenter et al. (11)	Recognition of strangulated small bowel obstruction	Low	Low	Low	High	High
Huang et al. (9)	Recognition of strangulated small bowel obstruction	High	Low	Low	High	High
BAR-N (14)	Strangulated bowel obstruction	Unclear	Low	Low	High	High
Xu et al. (13)	Strangulated bowel obstruction	Unclear	Low	Low	High	High
STRISK/NOFA et al. (14)	STRISK: strangulation; NOFA: failure of non-operative treatment	Low	Low	Low	Low	Low
Kim et al. (38)	Detection of small bowel obstruction on abdominal radiographs	Low	Low	Unclear	High	High
Oh et al. (19)	CT-based identification of high-risk acute small bowel obstruction	Unclear	Low	Unclear	High	High
Matsuda et al. (18)	CT-based identification of high-risk acute small bowel obstruction	High	Low	Unclear	High	High
Wang et al. (16)	Multimodal prediction of strangulation risk in adhesive small bowel obstruction	Unclear	Low	Low	Unclear	Unclear

Judgments were based on information reported in the original articles. “Low” indicates that the domain was adequately reported and unlikely to introduce major bias; “High” indicates clear methodological concerns; and “Unclear” indicates insufficient reporting to support a definitive judgment. Overall risk of bias was judged according to the highest level of concern across the four PROBAST domains.

The simplified PROBAST-based assessment showed that most existing prediction-model studies had at least unclear or high risk of bias, mainly in the analysis domain. Common limitations included retrospective single-center design, limited event numbers, insufficient external validation, incomplete reporting of missing-data handling, limited calibration assessment, and absence of decision-curve analysis. In contrast, studies with prospective multicenter design and external validation, such as STRISK/NOFA, had relatively stronger methodological credibility. Nevertheless, differences in clinical task, endpoint definition, patient population, imaging protocol, and validation design still limit direct comparison across models. These findings support the central interpretation of this review: reported AUC values should not be used alone to rank prediction models across heterogeneous studies.

## 7 Machine-learning, deep-learning, and multimodal AI models

Machine-learning models analyze structured clinical, laboratory, and imaging variables and may capture nonlinear effects that are difficult to represent in conventional regression. Algorithms such as LASSO and XGBoost are attractive because LASSO can reduce overfitting through penalized feature selection, whereas XGBoost can model nonlinear interactions and handle heterogeneous predictors. However, both can still overfit when sample sizes are small, events are rare, or hyperparameters are optimized without rigorous validation.

Deep learning is particularly relevant to image-based tasks. CNN-based models can learn patterns from abdominal radiographs or CT images without manual feature engineering. Recent studies have reported SFX-GAN, a sustainable and explainable multimodal spectral fusion framework for image dehazing in complex systems (39). Although developed in a different imaging context, this approach illustrates how artificial intelligence may improve image interpretation by enhancing image quality, increasing analytic efficiency, and strengthening model explainability and trustworthiness. Kim et al. reported high discrimination for detecting small bowel obstruction on plain radiographs, and later CT-based deep-learning studies explored automated identification of obstruction or high-risk patients. Multimodal models go further by combining EHR variables with CT-derived features. Such systems are conceptually powerful because they integrate clinical trajectory and anatomy, but they also require standardized data pipelines, image preprocessing, computational infrastructure, and prospective evaluation (40–42).

Machine-learning models have shown broad potential not only in the diagnosis and management of intestinal obstruction, but also in other clinical fields, including pancreatic cancer, lung cancer, diabetes, cardiovascular diseases, and brain tumors, among others (43–47). Their applications have further expanded beyond medicine to areas such as botany (48) and education (49). These cross-disciplinary applications suggest that artificial intelligence has particular strengths in processing complex, high-dimensional data and may support efficient clustering and pattern recognition through advanced algorithms, such as hybrid memetic bacterial intelligence-based methods (50).

Transformer-based and vision-transformer architectures are increasingly important in medical imaging because they can model long-range spatial relationships. For intestinal obstruction, they may help capture distributed bowel-loop patterns, mesenteric changes, and multi-slice CT context. However, transformers typically require large, well-annotated datasets and careful external validation; their use in intestinal-obstruction prediction should therefore be considered promising but not yet established.

Fusion strategies should be reported explicitly. Early fusion combines clinical variables and image-derived features before model training. Late fusion combines predictions from separate clinical and imaging models. Hybrid or attention-based fusion can learn how much weight to assign to each modality. Without this information, multimodal performance claims are difficult to reproduce or interpret.

Explainable AI methods such as Grad-CAM, LIME, and SHAP can improve transparency, but they should not be treated as proof of clinical validity. Grad-CAM can show whether an imaging model attends to plausible bowel or mesenteric regions, whereas SHAP can estimate the contribution of structured variables. Nevertheless, explanation maps may be unstable, and feature-attribution results can reflect dataset bias rather than causal mechanisms (51).

## 8 Critical comparison and methodological heterogeneity

Reported AUC values should be interpreted cautiously. A model predicting radiographic obstruction in a balanced image dataset is not directly comparable with a model predicting transmural necrosis in an emergency surgical cohort. Differences in disease prevalence, endpoint severity, inclusion criteria, CT protocol, surgical threshold, and validation design can make one AUC numerically higher without making the model clinically superior (Table 6).

**Table 6:** Structured comparison of methodological features, validation concerns, and implementation barriers across three categories of prediction models for intestinal obstruction.

Dimension	Clinical/laboratory scores	CT-integrated models	Multimodal AI models
Typical endpoint	Surgery, strangulation, or conservative-treatment failure	Strangulation, ischemia, necrosis, or need for operation	Detection, risk stratification, ischemia prediction, or multimodal decision support
Interpretability	High; variables are clinically intuitive	Moderate to high; CT signs are clinically meaningful	Variable; depends on XAI quality and clinician-facing interface
Data requirement	Low; routine bedside data	Moderate; CT availability and radiology expertise	High; imaging archives, EHR integration, annotations, compute resources
Validation concern	Often single-center and internally validated	May lack standardized CT interpretation or external validation	High risk of dataset shift, overfitting, and weak prospective evidence
Best use case today	Initial triage and low-resource settings	Radiology-supported surgical decision-making	Research or tertiary-center decision support after robust validation
Key implementation barrier	Sensitivity may be insufficient for rule-out decisions	Access to contrast-enhanced CT and radiologist variability	Infrastructure, privacy, cybersecurity, fairness, explainability, liability

External validation is the most important step for clinical credibility. Internal validation using bootstrap or cross-validation reduces optimism but cannot prove generalizability. External validation should include different hospitals, scanners, patient populations, operative thresholds, and time periods. Prospective impact studies are needed to determine whether model use reduces delayed surgery, unnecessary laparotomy, bowel resection, complications, mortality, length of stay, or cost.

## 9 Translational, ethical, and medico-legal considerations

Clinical deployment of AI models for intestinal obstruction is challenging because emergency surgical workflows are time-sensitive and multidisciplinary. A useful system must deliver predictions quickly, show the reasons for risk classification, integrate with radiology and EHR systems, and support rather than interrupt clinical decision-making. Alerts must be calibrated to avoid both alert fatigue and missed high-risk cases.

Data privacy and cybersecurity are essential when models connect to hospital information systems. Interfaces should follow standard clinical data formats where possible, restrict data access by role, maintain audit logs, and ensure secure transfer and storage. Federated learning may enable multicenter model development without centralizing patient data, but it still requires harmonized definitions, quality control, and governance.

Fairness and bias should be assessed because single-center datasets may underrepresent older patients, rural hospitals, patients with renal dysfunction who cannot receive contrast CT, or hospitals with limited surgical resources. A model trained in a tertiary center may perform differently in a community hospital. Reporting should therefore include subgroup performance when sample size permits.

Medico-legal responsibility remains unresolved. AI predictions should be presented as decision support rather than autonomous decision-making. Surgeons and radiologists need documentation of model version, input data, confidence, explanation output, and recommended clinical interpretation. Prospective studies should evaluate whether clinicians understand and appropriately act on predictions.

## 10 Future research directions

First, future studies should define endpoints before model development and separate diagnostic models from prognostic or treatment-decision models. Standardized outcome definitions for strangulation, reversible ischemia, irreversible necrosis, and failure of conservative treatment would improve comparability.

Second, model-development reports should follow TRIPOD or TRIPOD-AI principles, include calibration and decision-curve analysis, and avoid presenting internal validation as proof of generalizability (52).

Third, multicenter prospective validation is essential. The most clinically meaningful studies will test whether prediction tools change management and improve patient-centered outcomes such as time to surgery, bowel preservation, complication rates, mortality, length of stay, readmission, and cost.

Fourth, dynamic models should be explored. Current tools often use admission data only, but obstruction evolves over hours. EHR-linked systems could update risk estimates using trends in vital signs, pain, inflammatory markers, lactate, electrolytes, decompression output, and repeat imaging.

Fifth, explainable, privacy-preserving, and adaptive learning frameworks should be developed cautiously. Federated learning, model monitoring, and periodic recalibration may help maintain performance across hospitals, but these methods require governance and transparent reporting.

## 11 Limitations of this review

This review has several limitations. First, the search was based primarily on PubMed and may have missed relevant studies indexed only in other databases. Second, although the search and selection process was reported transparently, this article was designed as a narrative review with a semi-systematic search strategy rather than a full PRISMA-compliant systematic review. Third, the simplified PROBAST-based assessment was applied to representative original prediction-model studies rather than to all included references, because the review also included guidelines, reviews, mechanistic articles, and methodological background papers. Therefore, conclusions regarding methodological quality should be interpreted as a structured narrative appraisal rather than a formal systematic risk-of-bias synthesis.

## 12 Conclusion

Predictive modeling in intestinal obstruction has progressed from transparent bedside scores to CT-integrated models and emerging multimodal AI systems. This evolution reflects a broader movement toward data-supported emergency surgical decision-making. However, current evidence does not justify an unqualified claim that AI models are clinically superior. Many studies are retrospective, endpoint definitions differ, external validation is incomplete, and real-world workflow impact remains uncertain.

At present, traditional scores are most useful for rapid triage and low-resource settings, CT-integrated models are practical adjuncts for radiology-informed surgical decisions, and AI-based multimodal models are promising but require prospective validation, explainability, fairness evaluation, cybersecurity safeguards, and implementation studies (53). The ultimate goal is not simply to maximize AUC, but to deliver timely, trustworthy predictions that reduce delayed treatment, avoid unnecessary surgery, and improve patient outcomes.
